# Neighborhood Blighted Property Removal and 311 Calls for Non‐Emergency Services: A Test of a Marker of Social Control

**DOI:** 10.1111/gean.12286

**Published:** 2021-03-15

**Authors:** Katherine P. Theall, Christopher N. Morrison, Sara F. Jacoby, Amber Tucker, Maeve E. Wallace, Michelle C. Kondo, Charles C. Branas, Jeanette Gustat

**Affiliations:** ^1^ School of Public Health and Tropical Medicine Tulane University New Orleans LA USA; ^2^ Department of Epidemiology Mailman School of Public Health Columbia University New York NY USA; ^3^ Department of Epidemiology and Preventive Medicine School of Public Health and Preventive Medicine Monash University Melbourne Australia; ^4^ Department of Family and Community Health University of Pennsylvania School of Nursing Philadelphia PA USA; ^5^ Northern Research Station USDA‐Forest Service Philadelphia PA USA

## Abstract

Many studies have demonstrated that collective efficacy is associated with positive health outcomes, lower crime, and violence in urban communities, and residents’ emotional connection to their community. Remediation of blighted properties has been theoretically linked to increases in collective efficacy. The purpose of this study was to examine the impact of blighted property remediation on city non‐emergency 311 calls for public incivilities and deterioration, as potential markers of collective efficacy. We used a quasi‐experimental design to test whether 311 calls for service changed around remediated vacant lots in New Orleans, Louisiana, United States, many of which were left vacant after Hurricane Katrina in 2005. In six city neighborhoods eligible for blighted property remediation as part of a city program, 203 treated vacant lots were matched 1:3 without replacement to control lots that were eligible for but did not receive treatment. This yielded a total of 812 vacant lots partitioned within 48 months, or 38,976 lot‐months. Controls were in the same New Orleans neighborhoods as their matched treatment lots but were at least 250 feet away to minimize contamination. Overall difference‐in‐differences models detected postintervention declines in calls related to dumping and garbage, and slight but mostly non‐significant changes in calls between intervention and control lots in all but calls for dumping and vehicles. Blighted property remediation may have an impact on dumping and garbage, which is important. Despite being geographically specific, low‐cost and longitudinal, the nature of 311 calls and structural and historic factors at play in both the concentration of vacant properties in communities and residents’ willingness to call must be considered. Further analyses of changes in 311 data and additional qualitative inquiry are warranted to more fully determine the utility of these data.

## Introduction

Several decades of scholarship identifies that neighborhood social, physical, and environmental conditions are related to the health of individuals who live and spend time in those places (Shaw and McKay 1942; Diez Roux 2001; Sampson 2003). Over time, scientific attention has moved from demonstrating that these associations exist to understanding the processes by which neighborhood conditions affect health, and harnessing these mechanisms to affect neighborhood change to improve health and wellbeing (Macintyre and Ellaway 2003; Duncan and Kawachi 2018; Roux 2018). One feature of the neighborhood environment that has been linked to several health outcomes is social capital (Kawachi and Berkman 2000), and one key component of social capital is collective efficacy—defined as the ability to mobilize and realize common goals. Collective efficacy is a central theoretical mechanism linking neighborhoods and health because it integrates underlying processes related to collective aspects of neighborhood life, including social control and social cohesion (Sampson, Morenoff, and Earls 1999). Many studies have demonstrated that collective efficacy is associated with positive health outcomes and lower crime and violence in urban communities, as well as how neighborhoods engender attachment or residents’ emotional connection to their community (Comstock et al. 2010).

Despite its importance for understanding and addressing neighborhood health and safety, measuring collective efficacy is no small task. Survey‐based measures do exist (Raudenbush 2003), but data collection sufficient to gauge neighborhood collective efficacy requires access and engagement with representative samples of residents within often large neighborhood geographies. This approach is highly resource intensive and can introduce bias in many forms, including selection bias (respondents may differ systematically from non‐respondents), measurement bias (survey items may be inappropriate for or misinterpreted by specific populations), and aggregation bias (combining responses from many people within neighborhoods may ignore variation within these units). Consequently, the use of other, routinely collected administrative data to assess and interpret collective efficacy offers an attractive alternative which could prove more efficient, amenable to different levels of geographic specification, and which could be more easily employed for monitoring changes over time (O’Brien, Sampson, and Winship 2015). That assumes, however, that a source or sources of administrative data are adequate markers of collective efficacy.

The utility of 311 call for service data has been explored in previous research focused on urban social environments. For example, O’Brien (2015) examined urban 311 call report data from the public call line for non‐emergencies, as a measure of custodianship. Custodianship is a concept similar to collective efficacy in that it measures the practice of taking action to prevent or repair physical disorder in a public space. Custodianship deals specifically with the physical act of tending to public spaces. Unlike collective efficacy, custodianship does not integrate community investment and perceptions of residents’ ability to improve public spaces. Looking at 311 calls regarding public maintenance, such as graffiti and pothole remediation, the authors found that city service hotlines may be useful resources for providing insight on levels of resident community engagement, but also as a potential indicator of physical disorder, capturing issues such as private neglect (e.g., homeowners not keeping up their yards) as well as public nuisance (e.g., graffiti) (O’Brien, Sampson, and Winship 2015). These researchers also compared 311 data to self‐reported data and observed a strong correlation between calls and norms of collective efficacy (O'Brien 2016). Other work has also demonstrated the utility of 311 data for monitoring correlates of public disorder in Washington D.C. (Wheeler 2018), or as markers for civic participation and community engagement in New York (White and Trump 2018).

Despite these known correlations, 311 data can introduce important biases (Crawford 2013), including systematic underrepresentation of residents unwilling or unable to call and log a 311 report. Representativeness is often influenced by the extent to which residents trust the capacity and will of local government to act on their behalf and respond to a call for service or complaint in a just way. Given the historic structural exclusion and exploitation people of color in the United States through municipally enacted policies like mortgage redlining and discriminatory policing (Aaronson, Hartley, and Mazumder 2020; Jacoby et al. 2018; Zenou and Boccard 2000), the representativeness of such data may be a substantial concern in effected communities. Others have suggested that although 311 data may not be an ideal marker for political engagement or participation when interpreted absent of the context of neighborhood conditions, these data can be used as broader marker for the extent of service demands that residents make and expect of city governments (White and Trump 2018).

One cadre of neighborhood conditions—blighted or vacant land and buildings—has gained increased and significant attention for its public health impact. Vacant lots and abandoned buildings are noticeable signs of neighborhood disinvestment and have been directly linked to violence, fear, and further disinvestment (LaGrange, Ferraro, and Supancic 1992; Skogan 1992; Ross and Mirowsky 1999; Wei et al. 2005; Keizer, Lindenberg, and Steg 2008; Branas et al. 2018). These lots may impact the health of nearby residents through the environmental hazards posed by overgrown vegetation, dumping, and tenuous buildings and structures. Abandoned lots and buildings may create locations for harboring illegal firearms, public drunkenness, illegal drug trafficking, and other unwanted activities (Branas et al. 2011, 2016; Kondo et al. 2015). Vacant properties have further been shown to reduce community cohesion and increase trash and rodents (Garvin et al. 2013).

Neighborhoods with high levels of abandoned lots and buildings are associated with not only increased crime but also negative health outcomes such as drug‐dependence mortality (Hannon and Cuddy 2006), sexually transmitted disease rates (Cohen et al. 2000), premature mortality (Cohen et al. 2003), and psychophysiological distress (Hill, Ross, and Angel 2005). Remediation of these conditions has therefore been the target of efforts in multiple municipalities to decrease crime and improve health. The theoretic basis of these efforts is that remediation changes the urban landscape in a way that promotes residents’ willingness and confidence to act in the service of their own security (Newman 1995) and improves neighborhood functioning (Taylor, Gottfredson, and Brower 1984) which can enhance social engagement and well‐being (Freisthler, Merritt, and LaScala 2006). In fact, one of the most consistent pieces of evidence in support of effective environmentally focused, place‐based violence interventions comes from studies of housing and blight remediation of buildings and land (Kondo et al. 2018).

Given the theoretical link between remediation of blighted properties and increases in collective efficacy, the purpose of this study was to examine the impact of blighted property removal on city call data on public incivilities and property deterioration (O’Brien 2015). We used a quasi‐experimental design to test whether 311 calls for non‐emergency services changed around remediated vacant lots in New Orleans, Louisiana, United States, many of which were left vacant after Hurricane Katrina in 2005.

## Method

### Study design

This retrospective longitudinal study examined associations between a targeted blighted property remediation program implemented by the City of New Orleans, Louisiana, and 311 calls for non‐emergency services for 2013–2016. Our spatial units were 812 vacant lots which we operationalized as centroid points. We partitioned these data within 48 months; thus, our space‐time units of analysis were 38,976 lot‐months.

### Treatment and control conditions

In 2005, Hurricane Katrina caused considerable damage to much of the housing and land stock in the greater New Orleans area. Although the impacts of this natural disaster effected people and places across the city’s socioeconomic strata, the ability of local residents to repair or rebuild depended on available resources. Many lower income areas remain in disrepair. In 2014, the City of New Orleans implemented the Flight the Blight program, codified in City Ordinance Chapter 66. This legislation enabled the city to remediate and perform routine maintenance (i.e., mowing) on properties in six targeted neighborhoods by using municipal resources, on behalf of property owners, with the costs of services recorded on the owners’ tax bill. City staff members identified properties during neighborhood inspection, with a target of 200 lots per neighborhood given available resources. Beginning in 2014, inspectors gave citations to lots that were unoccupied (usually vacant with no structure) and that had one or more of the following violations: grass or vegetation growth higher than 18 inches tall; trash, debris, or evidence of illegal dumping; and/or growth of noxious vegetation, such as poison ivy. Owners were given 7 days to remediate their property or request a code‐enforcement hearing. On fewer than one quarter of all cited lots, complaints by neighbors about nuisance properties triggered a citation and notice of violation by the city.

**Figure 1:**
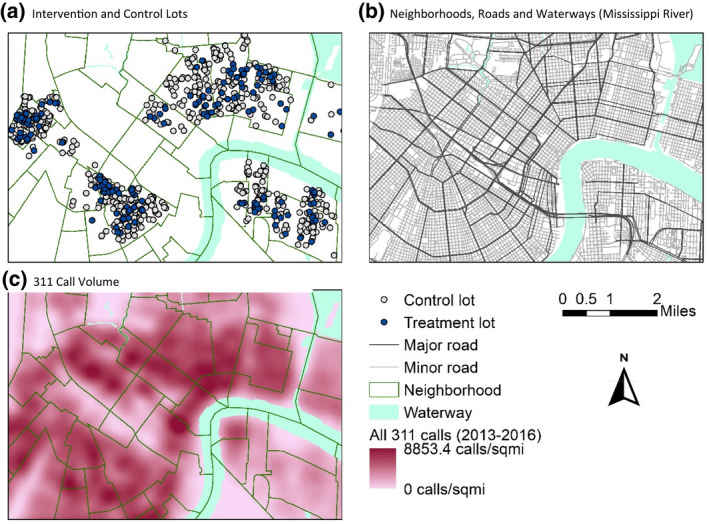
Example map showing 311 call density for all calls for January 2013 in New Orleans. [Colour figure can be viewed at wileyonlinelibrary.com]

From October 2014 to July 2016, White Dove Landscaping Service, which provided job training for at‐risk young people, remediated 1,614 properties in four large areas of the city (see Fig. 1) as part of a city‐contracted service. These areas exhibited not only a high volume of blighted property, but also high poverty, low education, low employment rates, and higher rates of violent crime. Lot remediation involved inspection, notice (including a letter to the owner and a sign placed on the property), and removal of debris and mowing of all grass. Lots were mowed at least once during the 2‐year period, and in some cases multiple times, with a frequency of no more than every 3 weeks, especially during summer months. New Orleans has a growing season that is almost year‐round, given the tropical climate.

### Matching

We defined treated lots as vacant lots that received debris removal and vegetation clearing under the Chapter 66 program. We identified 203 such lots, which we matched 1:3 without replacement to control lots that were eligible for but did not receive treatment. Controls were located in the same New Orleans neighborhoods as their matched treatment lots but were at least 250 feet away to minimize contamination.

### 311 calls data

As in other cities in the United States, the City of New Orleans maintains a registry of 311 calls for non‐emergency services. Available variables include the call type, date and time of the call, and the latitude and longitude for the requested service. We selected call types that were theoretically relevant for blighted property abatement and had at least 5,000 calls over the 4 study years. These primarily included calls arising from incivilities, although some may have been co‐classified as indicators of deterioration (O’Brien 2015). Included call types and call frequencies are presented in Table 1. The “other” call type includes all calls for which there were fewer than 5,000 calls over the 4 years, including calls for non‐functional streetlights, flooding, and potholes. We included these calls in the calculations for all calls.

**Table 1:** Frequencies of 311 Call Volume Within Categories; 2013–2016

Variable name	Call type	Calls	%
Dumping	Illegal dumping reporting	7,357	4.5
Garbage	Large item trash/garbage pickup	12,163	7.4
General	Code enforcement general request	25,219	15.4
Recycling	Residential recycling programs	17,432	10.6
Trash	Trash/garbage pickup	20,280	12.4
Trees	Tree service	7,070	4.3
Vehicles	Abandoned vehicle reporting/removal	15,460	9.4
n/a	Other	58,859	35.9
All	All calls	163,840	100.0

We used kernel density functions to produce spatially smoothed maps of the continuous densities of 311 calls per mile^2^ within call type categories for each calendar month. Fig. 1 shows an example for all calls for January 2013, with the treatment and control lots overlayed within New Orleans neighborhoods. The raster cell size was 108 × 108 feet, the raster extent was 27,021 × 44,639 feet, the search radius was set to the ArcGIS default, and decay was inverse distance weighted. We spatially joined the raster cell value for the 311‐call density for each calendar month to the treatment and control lots.

### Demographic characteristics

The demographic characteristics of residents may be associated with blighted property abatement and causally related to 311 calls and may therefore confound associations between blighted property abatement and 311 calls. We accessed American Community Survey data for 2011–2015 within Census tracts for median household income, percent of households at the federal poverty level, percent of people with a high school diploma, percent of unemployed and aged ≥ 16 years, and estimated percent of housing units that were vacant. To minimize the modifiable areal unit problem, we spatially joined the raster cell values to the treatment and control lot centroids. The resultant variables were time‐invariant estimates of the demographic characteristics at each treatment and control lot.

### Statistical analysis

The 311 data were zero truncated but otherwise normally distributed within lot‐months. We used Tobit regression models to account for bounding at a lower limit of zero, with sandwich estimators to account for the loss of unit independence due to geographic clustering of lots within matched groups. Our analysis used a difference‐in‐difference approach, in which we specified a treatment variable (1 for treated lots; 0 for control lots), and postintervention variable (1 for months on or after the intervention date for both treated lots and their matched controls; 0 otherwise), and a difference‐in‐difference term which is the product of these two variables. Models were adjusted for the inverse distance weighted demographic characteristics, New Orleans neighborhood, and a preintervention mean outcome interaction term to adjust for regression to the mean. For each call type we estimated five variants of these Tobit models. First, we included all lot‐month units to estimate the overall association between abatement and 311 calls during the study period. Next, we omitted lot‐months that were ≥ 4 months after the intervention, with the effect that the difference‐in‐difference term estimates the association between abatement and 311 calls within 3 months (1 quarter) after the intervention. We repeated this procedure to isolate associations for 4–6 months after the intervention (2 quarters), 7–9 after the intervention, and 10–12 months after the intervention.

## Results

There were 163,840 calls for 311 non‐emergency services in New Orleans for 2013–2016. Summary statistics for the geographic distribution of call types per mile^2^ and the demographic characteristics for the 39,168 lot‐months are presented in Table 2. On average, there were 82.7 calls per mile^2^ per month within the radius around all 812 treated and control lots.

**Table 2:** Summary Statistics for Lot‐Months; n = 39,168

Variable	Treatment lots (n = 9,792)		Control lots (n = 29,379)		*P* value
	Mean	SD	Mean	SD	
311 call density					
All	85.20	57.38	81.92	53.97	<0.001
Dumping	8.96	12.57	7.14	10.26	<0.001
Garbage	4.09	5.35	4.41	5.63	<0.001
General	20.53	20.62	17.54	18.62	<0.001
Recycling	5.60	6.86	6.11	7.15	<0.001
Trash	9.12	8.49	9.24	8.67	0.211
Trees	2.05	2.66	2.23	2.76	<0.001
Vehicles	8.49	13.90	9.16	12.21	<0.001
Demographic characteristics					
Median household income ($)	22,630.10	5,131.66	24,987.37	7,102.61	<0.001
Poverty (%)	41.65	8.04	38.89	8.31	<0.001
Unemployed (%)	16.07	4.24	15.07	3.83	<0.001
Education (%)	30.04	4.07	29.48	5.14	<0.001
Vacant housing (%)	25.83	4.42	24.85	4.46	<0.001

**Figure 2:**
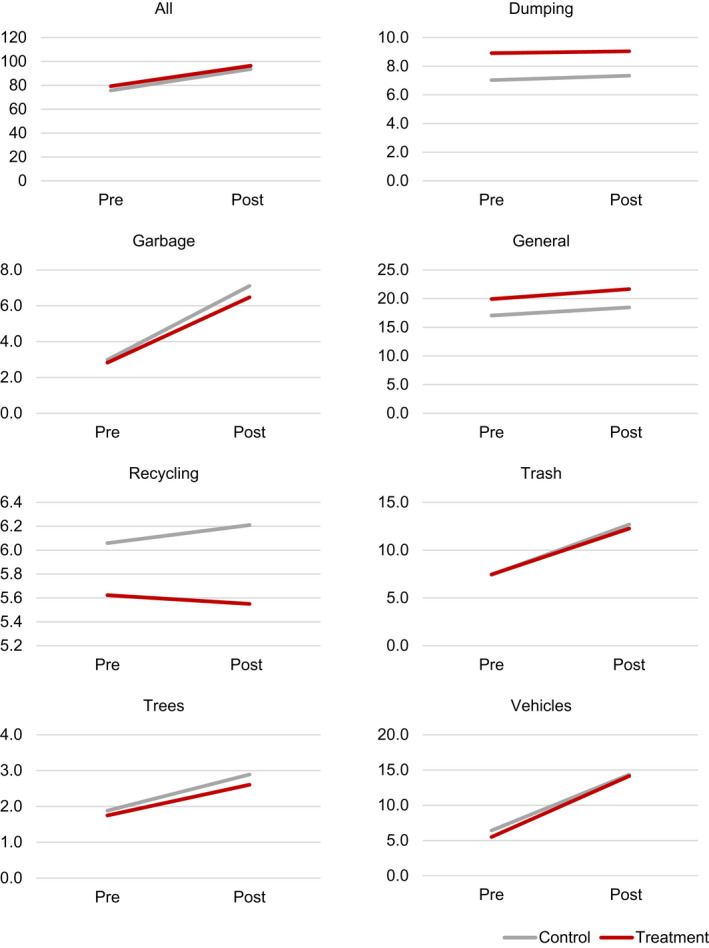
Mean 311 call density for treatment versus control lots and pre‐intervention versus post‐intervention months. [Colour figure can be viewed at wileyonlinelibrary.com]

Fig. 2 shows the mean call density per mile^2^ for 311 call types according for treatment versus control lots and preintervention versus postintervention months. The mean density of all calls for treatment lots was 79.3 per mile^2^ during preintervention months and 96.4 calls per mile^2^ during postintervention months. For matched controls, the mean density of all calls was 75.8 per mile^2^ during preintervention months and 93.6 per mile^2^ during postintervention months. Thus, the difference between preintervention and postintervention months was 17.1 calls per mile^2^ for treatment lots and 17.8 call per mile^2^ for matched controls, and the difference in these differences was −0.7 calls per mile^2^.

**Figure 3:**
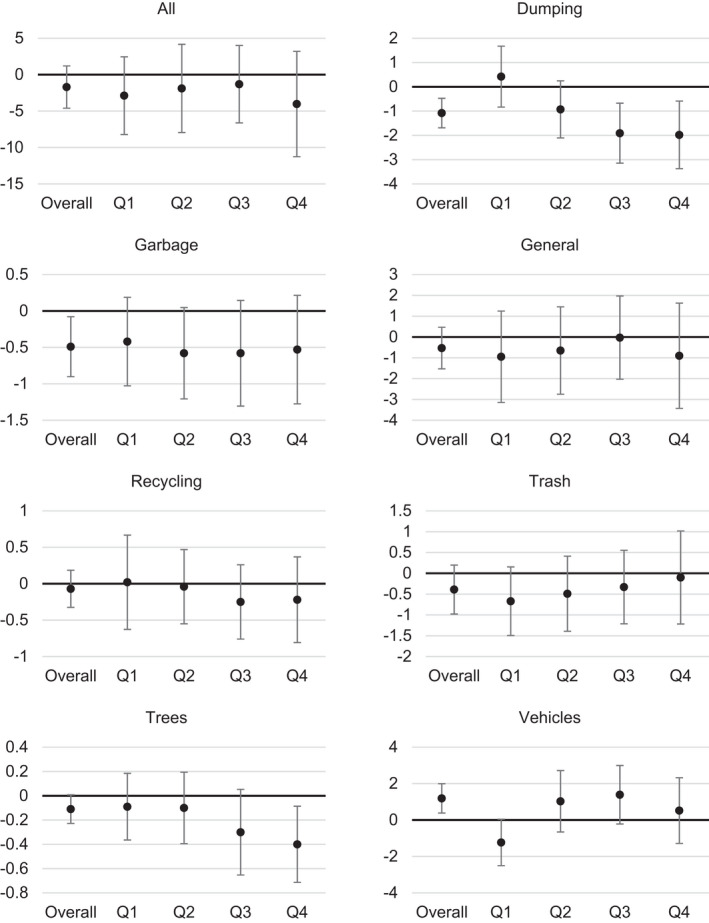
Post‐intervention effects over quarters (Qs) between treatment versus control lots and preintervention versus post‐intervention months. Overall denotes the association for all included months, Q1 is 1–3 months post‐intervention, Q2 is 4–6 months post‐intervention, Q3 is 7–9 months post‐intervention, and Q4 is 10–12 months post‐intervention.

Fig. 3 shows the parameter estimates for the difference‐in‐difference term in the Tobit regression models with adjustment for the population characteristics. The overall adjusted association for all calls is −1.7 calls per mile^2^, and the 95% confidence interval extends from a low estimate of −4.6 to a high estimate of 1.2. This interval includes the null value of 0.0. In quarters 1 to 4 (Q1–Q4), the point estimates are all negative and the confidence interval similarly includes the null value. The overall associations for dumping (b = −1.0, 95%CI: −1.6, −0.5) and garbage (b = −0.5; 95%CI: −0.9, −0.1) were negative, and the overall association for abandoned vehicles (b = 1.2; 95%CI: 0.4, 2.0) was positive. For dumping, these associations were strongest in Q3 and Q4, and for garbage the associations remained similar across the four quarters though the confidence intervals included the null value. For vehicles the point estimates switched from negative during Q1 (b = −1.2; 95%CI = −2.5, 0.0) to positive during Q2 to Q4 (e.g., Q2: b = 1.0; 95%CI: −0.7, 2.7), though in each quarter the confidence interval included the null value.

## Discussion

In this quasi‐experimental design, we examined the impact of blighted property remediation on 311 calls for non‐emergency services. Given the potential link between blighted properties in neighborhoods and collective efficacy, we hypothesized that collective efficacy would increase more around remediated vacant lots than those lots left vacant, and that this mechanism would manifest as increased incidence of 311 calls (Branas et al. 2011). We did not, however, find evidence of an increase in call volume for several types of complaints when comparing treatment and control lots. The level of calls increased over time (from 2013 to 2016) in both treatment and control areas and seemed to be primarily driven by calls for garbage, trash, vehicles, and trees.

Contrary to our expectation, the overall difference‐in‐differences models detected postintervention declines in calls related to dumping and garbage. After disaggregating by quarter, point estimates remained stable but the confidence intervals expanded (likely due to the smaller sample size) such that most associations included the null. The exception was dumping, which was associated with significantly fewer postintervention calls in quarters 3 and 4. Declines in calls related to dumping could be a result of blighted property removal, which may have led to declines in this activity or level of deterioration on select lots. The only significant association we found in the expected direction was for vehicles. The overall association for this call type was positive, though calls may have decreased in quarter 1 before climbing beyond their preintervention rates for quarters 2–4. This association may be due to an increase in parked cars on cleared land or an indicator of increased call volume to report vehicles.

Despite hypothesized relationships, trends suggest that treatment may have had a sustained effect and there was simply less to clean up in these areas. Furthermore, and echoing the sentiment that 311 data is not an ideal marker of political engagement or participation (White and Trump 2018), results do not necessarily suggest that the intervention did not impact collective efficacy, particularly given the limitations of 311 data. Some residents may not call 311 or may be uncomfortable or unempowered in doing so, especially in disadvantaged or disenfranchised communities given historical patterns of municipal investment (Massey and Hajnal 1995; Gibson 2007). Therefore, how well 311 calls reflect collect efficacy or social control is questionable.

Patterns of calls are often highly predictable (Xu et al. 2017), and many have linked 311 call volume to markers of socioeconomic status (SES) such as higher incomes (Feigenbaum and Hall 2015) and campaign donations (White and Trump 2018). However, others have found an inverse relation with SES markers, with higher 311 call volume in areas with greater socioeconomic decline such as foreclosure rate (Lacoe and Ellen 2014). During this study period, overall 311 call volume and calls for dumping, flooding, and trash were positively and significantly correlated with poverty rates in the neighborhoods, although the study area exhibited high poverty rates on average (approximately 40% of residents living below the U.S. federal poverty line, with a range of 10% to 80%) to begin with so are not representative of all neighborhoods. Looking at the hot spots for 311 calls in Fig. 1, areas not in this study but with high call volume are also areas with lower poverty rates. Call volume may also have differed by rental versus homeownership status (O’Brien 2015; Wheeler 2018; White and Trump 2018).

Although 311 data may not be the best marker for social control, it may still signal physical decay in a neighborhood (O’Brien, Sampson, and Winship 2015; Wheeler 2018) and it is important to note the observed declines in dumping and garbage in our study areas. 311 calls may be an indicator of sustained nuisances as opposed to 911 calls, which could signify “acute” nuisances. We have observed in our previous work (Branas et al. 2011) increases in police‐reported 911 disorderly conduct calls after vacant lot abatement, potentially an indicator of increased collective efficacy and desire by residents to protect newly greened spaces nuisances. Despite differences in these call types, the fact that remediation of vacant land may result in reductions of sustained nuisances like illegal dumping is important.

However, collective perception of what signifies decay or nuisances may vary by neighborhood, and reasons for calling the system may also reflect perception of the issue at hand rather than any change in engagement or social control. Both physical disorder and collective efficacy have been inversely associated with SES (Sampson, Raudenbush, and Earls 1997). However, others have found that areas with lower SES may exhibit greater levels of social capital (Coffé and Geys 2006). As discussed above, poverty rate was positively associated with 311 calls over the study period. Questions remain, however, as to what forces weaken or strengthen a community’s collective efficacy or social control. Although removal of blighted property is theoretically expected to improve efficacy, 311 calls may not be the ideal marker to capture such changes. Future work may consider pairing objective data with more qualitative, in‐depth exploration of these constructs and alternatives for measuring them, as well as data that explores group‐level connections within neighborhoods to accurately get a sense of efficacy in an area.

### Limitations

Despite a strong design and important results, our study had several limitations. First, as discussed, the generalizability of blighted property remediation in the tropical environmental conditions of a city like New Orleans is limited. Second, there may have also been other property remediation programs operating concurrently during the study period, given that the city was still recovering from Hurricane Katrina. Therefore, it is possible that our pre–post and case–control designations were contaminated, and this could have biased our results toward the null. We addressed this limitation, however, by selecting neighborhoods that were targeted by the city’s Fight the Blight program and a temporal period to minimize this problem. We attempted to validate the treatment effects of the Fight the Blight program using Google Street View to examine differences in the physical conditions sustained by lots over time. A third limitation is that post hoc validation of remediated lots was difficult because the years of availability and consistency across lots varied significantly. Fourth, in addition to the aforementioned limitations of 311 data, it may also be limited for discerning collective efficacy and residential engagement from intensive individual engagement. Finally, the number of times an individual can call 311 is not limited, and it is impossible to know how many residents actually called the system. It is possible that the data we used here primarily captures the engagement of super‐users (O’Brien, Sampson, and Winship 2015).

### Conclusions

Although blighted property remediation of vacant lots has the potential to impact community engagement and potential social control among residents, 311 data may not be the best marker to monitor such an effect. Despite being geographically‐specific, low‐cost, and longitudinal, the nature of 311 calls and structural and historic factors at play in both the concentration of vacant properties in communities and residents’ willingness to call city municipalities must be considered. Further analyses of changes in 311 data and additional qualitative inquiry are warranted to more fully determine the utility of these data.

## Conflict of Interest

There are no conflicts of interest to report.
